# The aberrantly expressed miR-193b-3p contributes to preeclampsia through regulating transforming growth factor-β signaling

**DOI:** 10.1038/srep19910

**Published:** 2016-01-29

**Authors:** Xinyao Zhou, Qiaoli Li, Jiawei Xu, Xiaojing Zhang, Huijuan Zhang, Yuqian Xiang, Chuantao Fang, Teng Wang, Shihui Xia, Qiang Zhang, Qinghe Xing, Lin He, Lei Wang, Mingqing Xu, Xinzhi Zhao

**Affiliations:** 1 Children’s Hospital and Institutes of Biomedical Sciences, Fudan University, 138 Yixueyuan Road, Shanghai 200032, China; 2 State Key Laboratory of Genetic Engineering and MOE Key Laboratory of Contemporary Anthropology, School of Life Sciences, Fudan University, Shanghai, China; 3 Department of Obstetrics, Provincial Hospital Affiliated to Shandong University, Jinan, China; 4 Departments of Pathology and Biobank, International Peace Maternity & Child Health Hospital of China affiliated to Shanghai Jiao Tong University, Shanghai, China; 5 Bio-X Institutes, Key Laboratory for the Genetics of Developmental and Neuropsychiatric Disorders (Ministry of Education), Shanghai Jiao Tong University, Shanghai, China

## Abstract

Preeclampsia (PE) is a leading cause of maternal mortality worldwide. Several studies have detected some differentially expressed microRNAs in the preeclamptic placenta, but few of the identified microRNAs demonstrated consistent findings among different research studies. In this study, high-throughput microRNA sequencing (HTS) of 9 preeclamptic and 9 normal placentas was performed. Seventeen microRNAs were identified to be up-regulated, and 8 down-regulated in preeclamptic placentas. Eight differentially expressed microRNAs except one identified in our study were determined to be consistent with at least one previous study, while sixteen were newly found. We performed qRT-PCR with independent 22 preeclamptic placentas and 20 control placentas to verify the differentially expressed microRNAs, and ten microRNAs were validated. The predicted target genes of the aberrantly expressed *miR-193b-3p* were enriched in the following gene ontology categories: cell motility and migration, cell proliferation and angiogenesis. We also found that *miR-193b-3p* significantly decreased the migration and invasion of trophoblast (HTR-8/SVneo) cells and that *miR-193b-3p* could regulate trophoblasts migration and invasion through binding onto the 3′UTR target site of *TGF-β2*. In conclusion, we identified a list of differentially expressed microRNAs in PE placentas by HTS and provided preliminary evidence for the role of *miR-193b-3p* in the pathogenesis of preeclampsia.

Preeclampsia (PE) is characterized by the de-novo development of hypertension and proteinuria at ≥20 weeks of gestation^1^. This pregnancy-specific syndrome complicates approximately 2% to 8% of pregnancies and is a leading cause of maternal mortality worldwide^2^. Delivery of the placenta is the only effective treatment for preeclampsia, indicating that the placenta is indispensable to the development of preeclampsia^3^. According to the traditional view of the pathogenetic mechanisms involved in preeclampsia, initial insults disrupt the deep invasion of the trophoblast resulting in shallow implantation and abnormal remodelling of the placental spiral artery long before 12 to 20 weeks of gestation, and in response to poor placentation, proinflammatory and antiangiogenic factors from the foetal/placental unit modify maternal physiology manifesting the clinical characteristics of PE^4^,^5^,^6^,^7^.

MicroRNAs(MiRNAs) are non-coding RNAs with approximately 21–25 nucleotides in length, which are estimated to post-transcriptionally regulate the expression of nearly 30% of all genes in animals by cleavage or translational interference^8^. MiRNAs are involved in regulating trophoblast proliferation, apoptosis, migration and invasion, and have been suggested to play an important role in the regulation of placental development and function^9^. Several studies based on hybridization, RT-qPCR or sequencing analysis have detected a list of differentially expressed miRNAs in PE placenta. However, only limited number of these identified miRNAs demonstrated consistent findings among these studies. For instance, six miRNA profiling research analyses showed that *miR-210* was aberrantly expressed^10^,^11^,^12^,^13^,^14^,^15^ and three showed that *miR-193b-3p* consistently up-regulated in PE placentas^10^,^11^,^12^. Efforts have been made to identify how differentially expressed miRNAs contribute to the onset of PE. Evidence suggested that dysfunction of miRNAs could inhibit migration and invasion of human extravillous trophoblast-derived HTR-8/SVneo cells^16^,^17^. However, it is not yet the right time to use these findings from miRNA studies on preeclampsia to improve the management or early recognition of this condition. Further investigation is required to elucidate novel mechanisms underlying the molecular pathology of PE by specific miRNAs which have not been investigated, like *miR-193b-3p*.

High-throughput sequencing (HTS) platforms perform well in reproducibility, accuracy, specificity and sensitivity for quantitatively measuring RNA. In addition, the detection sensitivity has been shown to be increased along with increasing sequencing depth, which is superior to hybridization-based platforms, whereas RT-qPCR is limited by the number of microRNAs^18^. Four studies conducted by Guo *et al.* 2011^19^, Ishibashi *et al.* 2012^12^, Weedon-Fekjær *et al.* 2014^20^ and and Yang *et al.* 2015^21^ also performed miRNA profiling of PE placentas using sequencing platforms. However, three^12^,^19^,^21^ performed the research based on limited sample sizes, and one^20^ conducted the research with limited sequencing depth.

In the present study, we performed HTS analysis on 9 preeclamptic and 9 normal placentas with enough sequencing depth to explore the differentially expressed miRNAs and further investigate their biological roles in the development of PE. Our study demonstrated that the expression of *miR-193b-3p* was significantly up-regulated in PE placentas, and it significantly decreased the migration and invasion of HTR-8/SVneo cells. Furthermore, we found that transforming growth factor-beta 2 (*TGF-β2*) was post-transcriptionally dysregulated by *miR-193b-3p* and confirmed that *miR-193b-3p* could regulate trophoblast cell invasion and migration by targeting *TGF-β2*.

## Results

### Characteristics of microRNA expression by HTS

**Figure 1:**
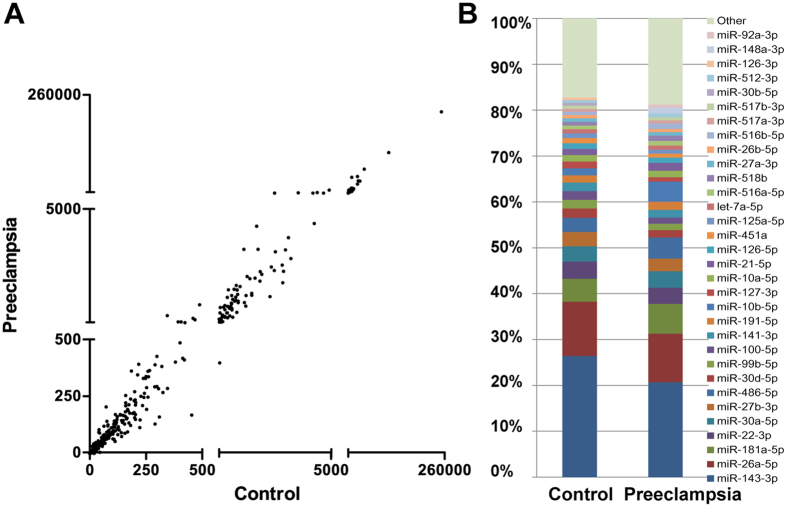
Characteristics of placental microRNA expression profiles in normotensive and preeclamptic pregnancy. Global placental microRNA expression distribution (sequence reads ≥5) of normotensive controls (n = 9) and preeclamptic patients (n = 9) by HTS (**A**). The top 30 microRNAs constitute ~80% of all differentially expressed microRNAs between control and preeclamptic placentas (**B**).

We performed HTS on 9 preeclamptic placentas (P1–P9) and 9 normal placentas (control group, C1–C9). We analyzed miRNAs with ≥5 sequence reads and constructed a scatter plot of these 787 miRNAs with the normalized reads (per million). Comparing global miRNA expression profiles in placentas of preeclamptic patients with those of normotensive controls, we could intuitively observe the distribution of expression of the two groups (Fig. 1A). The majority of miRNAs were not significantly differentially expressed between control and preeclampsia subjects, and the expression level of the majority of miRNAs was low (below 500 reads per million reads). Top 30 miRNAs constituted around 80% of all miRNA expression in normal and preeclamptic placenta tissues (Fig. 1B, Supplementary Table S1).

### Differentially expressed microRNAs in placentas of patients with preeclampsia

**Figure 2:**
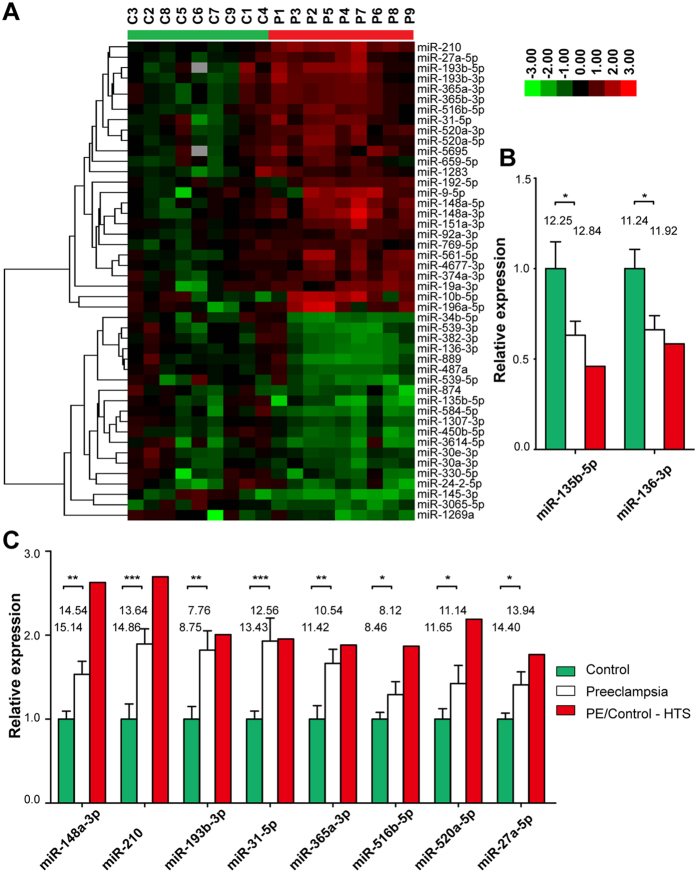
Hierarchical cluster analysis and validation of differentially expressed microRNAs. 46 differentially expressed microRNAs were selected using the following criteria: q value ≤0.05; fold change ≥1.5 or ≤0.66. The relative expression level was log transformed. Each column represents a placental sample from a pregnant control without preeclampsia (C1–C9) or with preeclampsia (P1–P10). The colour bar in the upper left indicates high expression (red) or low expression (green) (**A**). Validation of differentially expressed microRNAs by qRT-PCR. 2 down-regulated microRNAs (**B**) and 8 up-regulated microRNAs (**C**) are validated by qRT-PCR with independent control placentas (n = 20) and PE placentas (n = 22). The relative expression of each unique miRNA was normalized by the value of the U6 gene. We normalized the control as 1 by dividing each value by the average value of the control group. PE/Control–HTS indicates the fold change of PE placental microRNA expression level relative to the control as demonstrated by HTS. The mean raw ΔCT (CT_,miRNA_ − CT_,U6_) values of each miRNA were shown on top of the bars. Data are shown as the mean ± S.E.M. Significant differences were determined by 2-tailed Student’s t test or the Mann-Whitney test. ***P < 0.001, **P < 0.01, *P < 0.05.

A total of 25 miRNAs were significantly deregulated, 17 of which were up-regulated and 8 were down-regulated in preeclampsia (Table 1). Cluster analysis based on 46 selected microRNAs (p value ≤0.05; fold change ≥1.5 or ≤0.66) generated a tree with a clear distinction between the preeclampsia and control groups (Fig. 2A).

### Validation of differentially expressed microRNAs by qRT-PCR

Quantitative RT-PCR was carried out to validate the differentially expressed miRNAs in preeclampsia detected by HTS (Fig. 2B,C). Twelve miRNAs that demonstrated differential expressions in PE by HTS were selected for qRT-PCR validation based on independent 22 PE placentas and 20 control placentas. Ten miRNAs were successfully validated by qRT-PCR, including 8 up-regulated miRNAs (*miR-148a-3p, miR-210, miR-193b-3p, miR-31-5p, miR-365a-3p, miR-516b-5p, miR-520a-5p* and *miR-27a-5p*) and 2 down-regulated miRNAs (*miR-135b-5p* and *miR-136-3p*). Additional 2 miRNAs (*miR-10b-5p*, and *miR-192-5p*) were not validated in these independent samples.

### Consistency of the identified differentially expressed microRNAs between the present study and previous reports

Using a combination of the differentially expressed microRNAs from our study and the results based on previous array analysis, we identified a complete list of 182 differentially expressed miRNAs. Forty-four differentially expressed miRNAs are present in Supplementary Table S2. These miRNAs were identified in at least two studies, and most of the studies on the corresponding miRNAs reported consistent findings. *MiR-210*, which was reported in 8 studies, was the most common recurring miRNA among the 44 differentially expressed miRNAs. Indeed, the function of *miR-210* in preeclampsia has also been widely investigated compared to other miRNAs^12^,^22^,^23^. The second most common recurring miRNA was *miR-193b-3p,* which was presented in 4 studies and consistently showed up-regulation in preeclampsia. However, to our knowledge, no research on the function of *miR-193b-3p* in preeclampsia has been conducted to date. Although there are additional 2 miRNAs (*miR-181a-5p* and *miR-363-3p*) present in 4 studies, they were not found to be differentially expressed in our study. Focused on our own study, 8 differentially expressed miRNAs (*miR-210*, *miR-193b-3p, miR-31-5p, miR-193b-5p, miR-10b-5p, miR-151-3p, miR-520-3p* and *miR-584-5p*) were found to be consistent with at least one previous study. One miRNA, *miR-192-5p,* which demonstrated differential expression in our HTS analysis, was also present in two previous studies with the opposite findings. It should be noted that *miR-192-5p* showed no differential expression in our qRT-PCR validation in an independent cohort. The remaining differentially expressed miRNAs (16 miRNAs) from our study were newly found.

### Effect of *miR-193b-3p* on HTR-8/SVneo migration and invasion

We focused on microRNAs that demonstrated consistent findings among a number of independent studies. *MiR-193b-3p* was the second most common recurring miRNA after *miR-210*. We inferred that *miR-193b-3p* might have an important role in the progression of preeclampsia.

**Figure 3:**
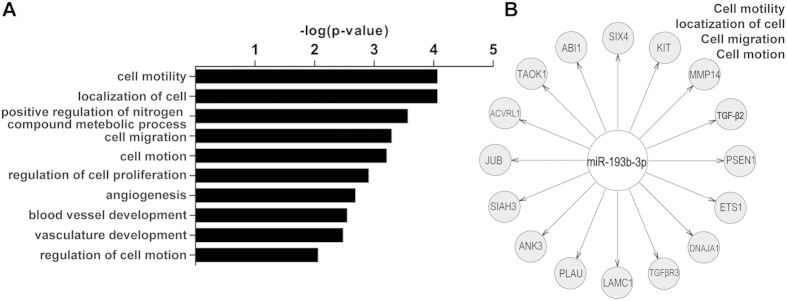
Gene ontology (GO) analysis of predicted targets of *miR-193b-3p.* Gene ontology analysis of predicted 221 targets of *miR-193b-3p* (predicted by TargetScan) was performed on DAVID (**A**). The most significantly enriched functional annotation cluster of the GO analysis is cell motility, cell migration and cell motion (**B**).

We found that the genes predicted to be targets of *miR-193b-3p* by TargetScan were enriched in the following gene ontology categories: cell motility and migration, cell proliferation, positive regulation of nitrogen compound metabolic process and angiogenesis (Fig. 3A). In addition, we noticed that the most significantly enriched functional annotation cluster among these 221 targets of *miR-193b-3p* was cell motility, cell migration and cell motion (Fig. 3B). This result suggests that *miR-193b-3p* directly or indirectly regulates genes involved in cell migration.

**Figure 4:**
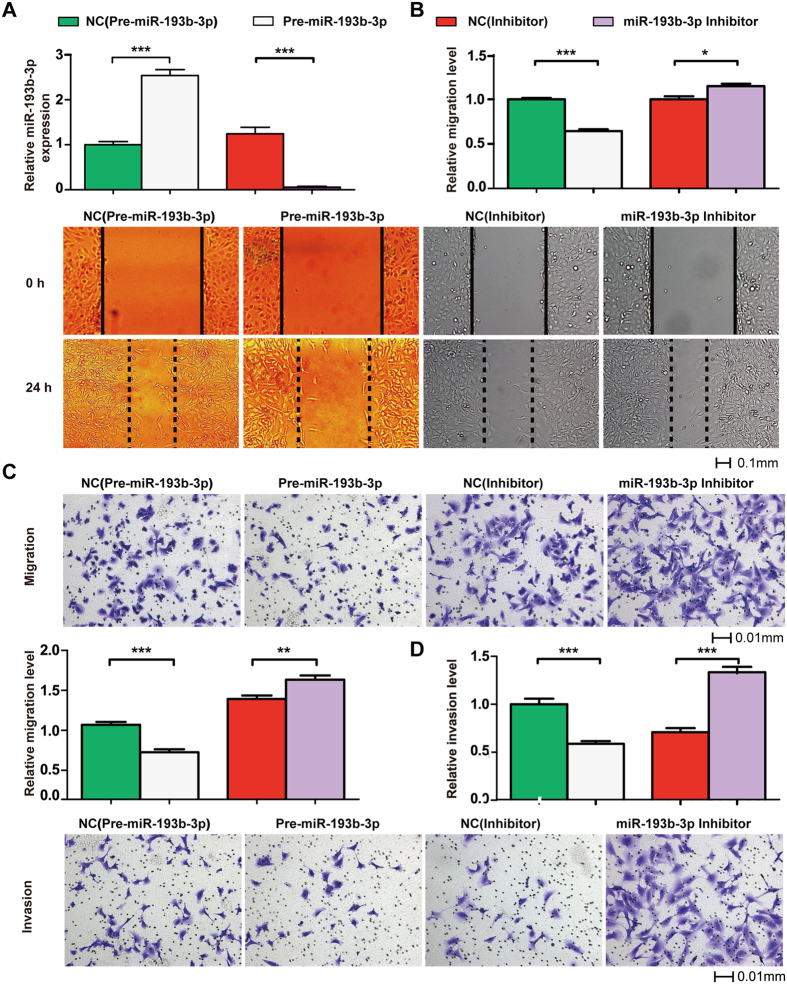
Function study of *miR-193b-3p* in preeclampsia. We evaluated the efficiency of Pre-miR-193b-3p and *miR-193b-3p* inhibitor (**A**). Trophoblast (HTR-8/SVneo) cells were transiently transfected with pre-miR-193b-3p, *miR-193b-3p* inhibitor or the corresponding control constructs (negative control, NC). The migration ability was assessed 24 h from the scratch (**B**). To investigate the effect of *miR-193b-3p* overexpression, 54 scratch spots were assessed for either Pre-miR-193b-3p group or NC pre-miR-193b-3p group. To investigate the effect of *miR-193b-3p* inhibition, 10 scratch spots were assessed for each experiment group. The transwell migration assay was assessed 6 h from plating cells (**C**). The transwell invasion assay was assessed 24 h from plating cells (**D**). The number of invaded cells was counted in 5 different fields of each membrane under light microscope. All experiments were repeated three times. Data are shown as the mean ± SEM. Significant differences were determined by Student’s t test. ***P < 0.001, **P < 0.01.

We then evaluated the effects of *miR-193b-3p* on the migration of a trophoblast (HTR-8/SVneo) cell line with *in vitro* scratch assay (Fig. 4B) and transwell migration assay (Fig. 4C). We evaluated the efficiency of *miR-193b-3p* overexpression constructs (Pre-miR-193b-3p) and *miR-193b-3p* inhibitor (Fig. 4A). The fold-change of relative *miR-193b-3p* expression after transfecting Pre-miR-193b-3p in HTR-8/SVneo was 2.54. This fold-change and the relative *miR-193b-3p* expression in HTR-8/SVneo were similar to the result from our miRNA profiling of PE and control placentas by HTS. Transfecting *miR-193b-3p* inhibitor decreased the expression of *miR-193b-3p* in HTR-8/SVneo by 95%. As shown in our *in vitro* scratch assay, *miR-193b-3p* significantly decreased the migration of HTR-8/SVneo by 36% compared to the corresponding negative control (*P* < 0.001, Fig. 4B) and inhibition of *miR-193b-3p* significantly improved the migration of HTR-8/SVneo by 15% compared to the corresponding negative control (*P* < 0.05, Fig. 4B). As shown in transwell migration assay, *miR-193b-3p* significantly decreased the migration of HTR-8/SVneo by 37% compared to the corresponding negative control (*P* < 0.001, Fig. 4C) and inhibition of *miR-193b-3p* significantly improved the migration of HTR-8/SVneo by 19% compared to the corresponding negative control (*P* < 0.01, Fig. 4C).

We also assessed the effects of *miR-193b-3p* on the invasion of a trophoblast (HTR-8/SVneo) cell line with transwell invasion assay. *MiR-193b-3p* significantly decreased the invasion of HTR-8/SVneo by 41% compared to the corresponding negative control (*P* < 0.001, Fig. 4D), and inhibition of *miR-193b-3p* significantly improved the invasion of HTR-8/SVneo by 88% compared to the corresponding negative control (*P* < 0.001, Fig. 4D).

### *TGF-β2* was identified as a direct target of *miR-193b-3p*

We re-investigated the results of our previous study concerning protein-coding gene expression profiles in PE placentas^24^. We found that *TGF-β2* was the only differentially expressed gene among the 16 predicted *miR-193b-3p* targets involved in the functional annotation cluster of cell motility, cell migration and cell motion (Fig. 3B).

**Figure 5:**
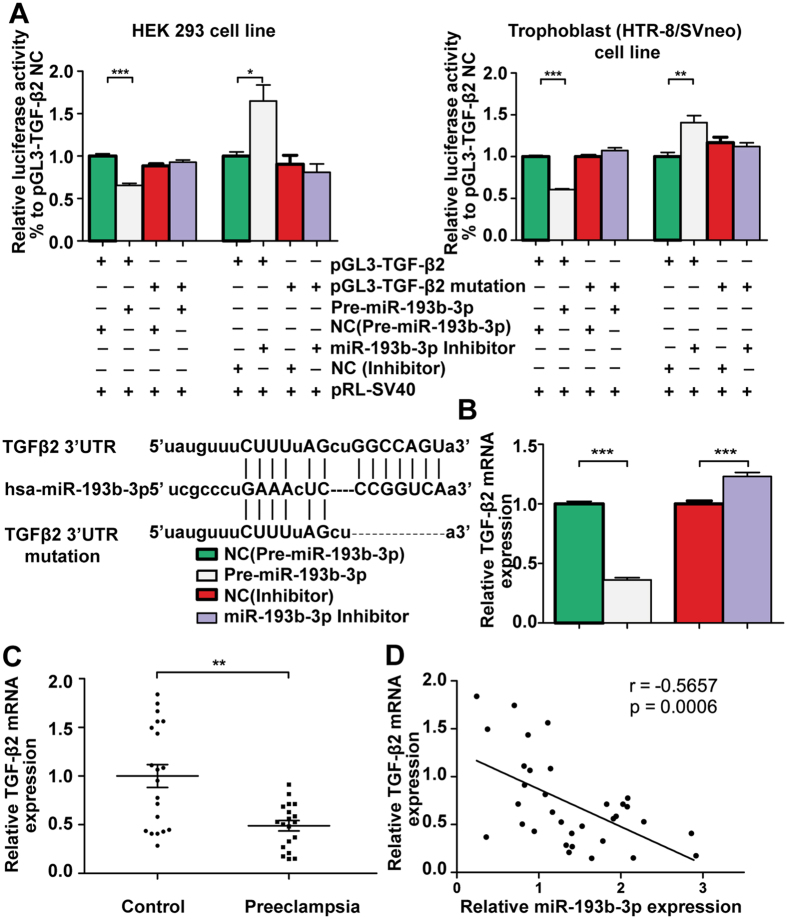
Validation of *TGF-β2* as the direct target of *miR-193b-3p.* The pGL3 Luciferase Reporter Vector (pGL3-TGF-β2 or pGL3-TGF-β2 mutation) were cotransfected with the *miR-193b-3p* precursor (Pre-miR-193b-3p), *miR-193b-3p* Inhibitor or the corresponding control constructs (negative control, NC) into 293 and trophoblast (HTR-8/SVneo) cell lines. Luciferase activity was measured (**A**). Sequence alignment between the normal and mutated sequence of *TGF-β2* 3′-UTR and *miR-193b-3p* was showed in the lower channel. When constructing the mutation type, 7 bp of the wild TGFβ2 3′UTR which matched the seed sequence of *miR-193b-3p* was deleted. (**A**). HTR-8/SVneo cells were transfected with the *miR-193b-3p* precursor (Pre-miR-193b-3p), *miR-193b-3p* Inhibitor or the corresponding control constructs (negative control, NC), and the *TGF-β2* mRNA level was assessed (**B**). *TGF-β2* mRNA was significantly down-regulated in PE placentas (**C**) and was negatively correlated with the expression of *miR-193b-3p* (**D**).

To analyse whether *miR-193b-3p* directly binds to the predicted conserved binding site, we cloned the full length of *TGF-β2* 3′UTR into the pGL3 Luciferase Reporter Vector (pGL3-TGF-β2), and the same *TGF-β2* 3′UTR with a mutation at the corresponding conserved *miR-193b-3p* binding site was also cloned into the pGL3 Luciferase Reporter Vector (pGL3-TGF-β2 mutation). Relative luciferase activity of constructs with wild type *TGF-β2* 3′UTR in 293 cell line was reduced to 65% (*P* < 0.001, Fig. 5A) when overexpressing *miR-193b-3p,* and was increased to 164% (*P* < 0.05, Fig. 5A) when inhibiting *miR-193b-3p;* whereas a change in luciferase activity was not observed in the construct with mutant *TGF-β2* 3′UTR. Meanwhile, relative luciferase activity of constructs with wild type *TGF-β2* 3′UTR in HTR-8/SVneo was reduced to 60% (*P* < 0.001, Fig. 5A) when overexpressing *miR-193b-3p*, and was increased to 141% (*P* < 0.01, Fig. 5A) when inhibiting *miR-193b-3p*; whereas a change in luciferase activity was not observed in the construct with mutant *TGF-β2* 3′UTR. We confirmed that *miR-193b-3p* could repress *TGF-β2* expression by directly binding to a conserved 3′UTR site of *TGF-β2*.

We also assessed the *TGF-β2* mRNA level in HTR-8/SVneo cells after transfection with *miR-193b-3p* precursor (Pre-miR-193b-3p), *miR-193b-3p* Inhibitor or control constructs (negative control, NC). *miR-193b-3p* significantly decreased *TGF-β2* mRNA level in HTR-8/SVneo cells by 64% compared to the corresponding negative control (*P* < 0.001, Fig. 5B) and inhibition of *miR-193b-3p* significantly improved the expression of *TGF-β2* mRNA level in HTR-8/SVneo by 23% compared to the corresponding negative control (*P* < 0.001, Fig. 5B).

We then measured *TGF-β2* levels by qRT-PCR in all placental tissues and found that *TGF-β2* mRNA was significantly down-regulated in PE placentas (*P* < 0.01, Fig. 5C). A significant negative correlation between the expression of *miR-193b-3p* and *TGF-β2* mRNA was observed (*P* < 0.001, Fig. 5D). We also found that the TGF-β2 protein level was approximately 65% lower in PE placentas (Supplementary Fig. S1).

### The function of *miR-193b-3p* was exerted via regulation of *TGF-β2*

**Figure 6:**
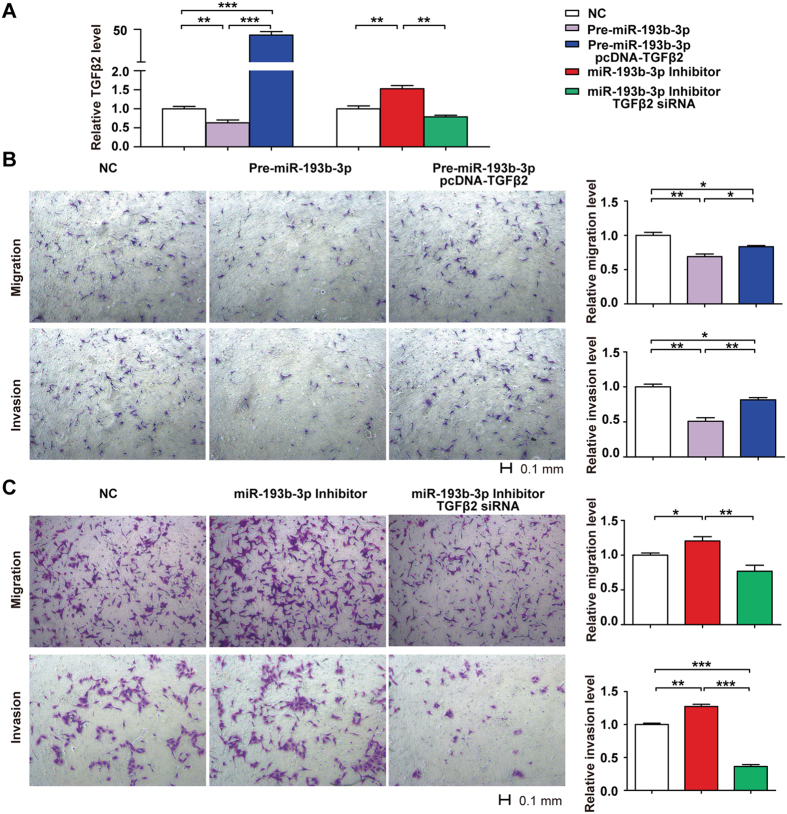
Restoring *TGF-β2* expression reverses the regulation of *miR-193b-3p* on Trophoblast (HTR-8/SVneo) cells migration and invasion. Simultaneous transfection of HTR8/SVneo cells with the *miR-193b-3p* inhibitor and *TGF-β2* siRNA reduced the ability of the *miR-193b-3p* inhibitor to upregulate *TGF-β2* mRNA expression at 48 h after transfection and co-transfection of pcDNA-TGFβ2 restored *TGF-β2* expression (**A**). Overexpression of TGF-β2 (pcDNA-TGFβ2) attenuates the functions of *miR-193b-3p* to inhibit cell migration and cell invasion at 48 h after transfection (**B**). Co-transfection of *TGF-β2* siRNA reduced the ability of *miR-193b-3p* inhibitor to promote cell migration at 48 h after transfection (**C**). Co-transfection of *TGF-β2* siRNA abolished the increased cell invasion promoted by transfection of the *miR-193b-3p* inhibitor at 48 h (**C**).

To verify whether the function of *miR-193b-3p* would be exerted via regulation of *TGF-β2*, we determined whether it could be possible to rescue *miR-193b-3p* inhibiting effect by TGF-β2 overexpression in HTR8/SVneo cells. Overexpression of TGF-β2 partially attenuated the inhibitory effects of *miR-193b-3p* on cell migration (P = 0.025, Fig. 6B) and cell invasion (*P* < 0.01, Fig. 6B) at 48 h after transfection.

In addition, simultaneous transfection of HTR8/SVneo cells with the *miR-193b-3p* inhibitor and *TGF-β2* siRNA reduced the ability of the *miR-193b-3p* inhibitor to upregulate *TGF-β2* mRNA expression at 48 h after transfection (*P* < 0.01, Fig. 6A). Furthermore, the transwell migration assay revealed that co-transfection of *TGF-β2* siRNA significantly reduced the ability of the *miR-193b-3p* inhibitor to promote cell migration at 48 h after transfection (*P* < 0.01, Fig. 6B). Additionally, the transwell invasion assay demonstrated that co-transfection of *TGF-β2* siRNA abolished the increased cell invasion promoted by transfection of the *miR-193b-3p* inhibitor at 48 h (*P* < 0.001, Fig. 6C). These findings indicated that *miR-193b-3p* could suppress HTR8/SVneo trophoblast cell migration and invasion through regulating *TGF-β2*.

## Discussion

In the present study, we detected a list of differentially expressed microRNAs in PE placentas by HTS. Eight differentially expressed miRNAs from our study were found to be consistent with at least one previous study and 16 microRNAs were newfound. However, only limited number of miRNAs demonstrated consistent results among different research studies. The inconsistency might be caused by the different clinical characteristics of patients enrolled in these different studies. This also might be due to the application of different experiment platforms and different sample sizes^18^,^25^. Although our sample size is relatively larger than previously published studies on the same topic, the small cohort may still be a limitation of the present study. Different experiment platforms have apparent differences in performance in reproducibility, accuracy, detection sensitivity and specificity^18^. Our follow-up study was focused on miRNAs that demonstrated consistent results among a number of independent studies.

For instance, *miR-210* is the most commonly identified differentially expressed miRNA in placental. Indeed, *miR-210* is one of the most investigated miRNAs in PE. It is a hypoxia-responsive miRNA involved in trophoblast migration and invasion, siderosis of interstitial trophoblasts and placental mitochondrial dysfunction^22^,^26^,^27^. Up-regulation of placental *miR-210* was also detected in our research.

*MiR-193b-3p* is the second most common recurring miRNA after *miR-210*. The findings in 3 previous studies and ours are consistent, demonstrating that *miR-193b-3p* is up-regulated in preeclamptic placentas. Previous publications suggested that *miR-193b-3p* was a putative tumor suppressor. This gene was found to be greatly down-regulated in several types of malignant tumors^28^,^29^. Moreover, overexpression of *miR-193b-3p* repressed cell proliferation and inhibited cancer cells invasion, migration and growth^29^,^30^. Typically, this was reported in the tumour microenvironment, but what about the placental environment? During placental development, cytotrophoblasts proliferate to form anchoring villi and give rise to interstitial trophoblasts that invade the uterine decidua and endovascular trophoblasts that migrate into the maternal spiral arteries; accordingly, the trophoblasts’ capacity for proliferation, migration and invasion plays a crucial role in successful placental development^31^. It appears that trophoblasts and cancer cells share similar biological characteristics in their proliferative, migratory and invasive properties^32^,^33^. Adequate invasion of tumour cells leads to cancer, but inadequate invasion of trophoblasts might result in poor placentation, leading to preeclampsia^34^. Consequently, the tumour suppressor role of *miR-193b-3p* is suggestive of its crucial role in the regulation of some principal trophoblasts events, leading to preeclampsia. In silico target prediction of *miR-193b-3p* by TargetScan and the following GO analysis showed that targets genes of *miR-193b-3p* were enriched in cell motility and cell migration. In addition, our *in vitro* scratch assay and transwell migration and invasion assays demonstrated that overexpression of *miR-193b-3p* significantly decreased the migration and invasion of HTR-8/SVneo cells, while inhibition of *miR-193b-3p* significantly improved the migration and invasion of HTR-8/SVneo cells. Our results indicated that the aberrant expression of *miR-193b-3p* in the preeclamptic placenta could contribute to the pathogenesis of preeclampsia through its inhibitory effect on the migration and invasion of trophoblasts.

We also showed that transforming growth factor-beta 2 (*TGF-β2*), a member of the transforming growth factor-β (TGFβ) superfamily, was post-transcriptionally dysregulated by *miR-193b-3p*. And restoring *TGF-β2* expression reversed the regulation of *miR-193b-3p* on trophoblast (HTR-8/SVneo) cells migration and invasion. Meanwhile, *TGF-β2* mRNA and protein expression were significantly decreased in preeclamptic placentas. According to previous research, the TGFβ signalling pathway is crucial to placental biological processes^35^. It is involved in regulating a number of cellular processes, including growth, migration, invasion, epithelial to mesenchymal transition (EMT) and immune reactions^36^,^37^. For instance, the cells undergoing EMT manifest a migratory phenotype and acquire invasive properties^38^, and the differentiation of interstitial trophoblasts and endovascular trophoblasts is considered to be an EMT-like transformation process^39^. Previous studies suggest that TGFβ is potent inducer of EMT in various cells^40^,^41^ and showed that the TGFβ family induced the invasive properties of a trophoblast cell line *in vitro*^42^. And one study showed that cell migration was improved by inducing *TGF-β2* expression under hypoxia^43^. In addition, soluble endoglin (sEng), which is well known to be involved in preeclampsia, was also identified as a TGFβ inhibitor in previous studies^44^,^45^. Therefore, we inferred that aberrant TGFβ signalling in the placenta might play a key role in the pathogenesis of PE; aberrant expression of *miR-193b-3p* in preeclamptic placentas could exert an inhibitory effect on the migration and invasion of trophoblasts by directly targeting *TGF-β2*. The detailed mechanism remains to be elucidated.

In conclusion, we detected and verified a list of differentially expressed microRNAs in PE placentas by HTS and qRT-PCR, and provided preliminary evidence for the role of *miR-193b-3p* in the pathogenesis of preeclampsia by targeting *TGF-β2*.

## Methods

### Sample collection

All placenta samples used in this study were collected from normal pregnant woman who were Han Chinese in origin (control group; n = 29) and from preeclamptic pregnant patients (PE group; n = 31) by elective caesarean delivery in the absence of labour during the third trimester of gestation (Table 2, All results are presented as the mean ± S.D.; BMI, indicates body mass index; DBP, diastolic blood pressure; and SBP, systolic blood pressure). Detailed clinical characteristics of patients with 9 normal and 9 preeclamptic pregnancies in HTS study were shown in Supplementary Table S3. PE was defined as systolic blood pressure (SBP) ≥140 mmHg and/or diastolic blood pressure (DBP) ≥90 mmHg on 2 occasions at least 4 hours apart with proteinuria ≥300 mg/day from 24 h urine collection occurring after the 20th week of gestation but resolving by the 12th week postpartum. All clinical placentas were collected immediately after the caesarean section. The placental samples at the chorionic plate were separately taken from each quadrants along with central portion in the placenta disc. ~1 cm^3^ fragments were dissected from the placenta, after removal of maternal blood by vigorous washing in phosphate buffered saline (PBS). The tissues were maintained in centrifuge tubes with RNAlater (Ambion Inc., Austin, TX), and then frozen at −80 °C. All the patients did not accepte antihypertensive medication and other special medical treatment before termination of pregnancy. All women enrolled in the study gave their written informed consent to the sample collection and analyses. This research was approved by the Institutional Review Committee of Institutes of Biomedical Sciences at Fudan University. All experiments were performed in accordance with relevant guidelines and regulations.

### MiRNA isolation and sequencing analysis

Total RNA was extracted using the Ambion mirVana™PARIS™Kit (Ambion , Carlsbad, California, USA) according to the manufacturer’s instruction. RNA concentration and quality were determined with Agilent 2100 Bioanalyser (Agilent, Palo Alto, CA). 1 ug total RNA was obtained for the small RNA sequencing analysis and underwent further processing with the TruSeq Small RNA Sample Preparation Kit. Briefly, 1 ug total RNA underwent adapter ligation, reverse transcription, PCR amplification and pooled gel purification to generate a library product. Following this, we performed microRNA-sequencing using the Illumina HiSeq 2000 sequencing system with approximately 6 multiplexed samples in each sequencing lane. The raw Illumina reads were pre-processed and mapped. The number of mapped reads was an average of 25.8 million in the control group and 28.4 million in the PE group. Using R version 2.14.0 statistical software, we screened the differentially expressed microRNAs based on the following criteria: p value ≤0.01; fold change ≥1.5 or ≤0.66.

### Validation of mRNA and miRNA by qRT-PCR

A total of 500 ng RNA was isolated for qRT-PCR validation. We performed real-time quantification of miRNAs by stem–loop RT–PCR based on previous studies^46^. The primers used are shown in supplementary table S4. Briefly, reverse transcriptase reactions were conducted with a total of 12.5 ul comprised of RNA samples, stem–loop RT primer, AMV Reverse Transcriptase (Promega, Madison, Wisconsin, USA), 5 × RT buffer (Promega, Madison, Wisconsin, USA), dNTPs (Promega, Madison, Wisconsin, USA) and RNase inhibitor (Promega, Madison, Wisconsin, USA). Real-time PCR was performed with samples containing RT product, microRNA-specific forward primer, universal reverse primer, ProbeLibrary Probe #21 (Roche, Penzberg, Germany) and FastStart Universal Probe Master (Rox) (Roche, Penzberg, Germany) using the Applied Biosystems 7900HT PCR system. RNU6B (U6) was used to normalize the microRNA qRT-PCR data.

A total of 500 ng RNA was isolated for mRNA qRT-PCR. The cDNA was synthesized using AMV Reverse Transcriptase system (Promega, Madison, Wisconsin, USA). *TGF-β2* mRNA qRT-PCR was performed using the Applied Biosystems 7900HT with FastStart Universal SYBR Green Master (Rox) (Roche, Penzberg, Germany). The primers for *TGF-β2* mRNA qRT-PCR are described in Supplementary Table S4.

The qRT-PCR data was analysed using the ΔΔCt method.

### Prediction of *miR-193b-3p* targets and GO analysis

We predicted targets of *miR-193b-3p* with only evolutionary conserved sites by TargetScan (TargetScanHuman 6.2), and 221 targets of *miR-193b-3p* were included. And then, we performed gene ontology(GO) analysis by use of DAVID (http://david.abcc.ncifcrf.gov/).

### Literature Review

We performed a search of literature published to October 2014 through PubMed to identify all articles reporting the differential expression of miRNAs in preeclampsia. In the 80 identified studies by using the the keywords “microrna” and “preeclampsia”, 33 were studies of human preeclamptic placentas compared to normotensive controls, which included 12 array studies based on hybridization, RT-qPCR or sequencing analysis that presented the results as a list of differentially expressed miRNAs^10^,^11^,^12^,^13^,^14^,^15^,^19^,^21^,^47^,^48^,^49^,^50^,^51^,^52^,^53^ and 21 studies that focused on specific miRNAs.

### Plasmid construction

A 119 base-pair DNA fragment encompassing the has-mir-193b gene was amplified with PCR from genomic DNA by use of the following primers: forward, 5′-TCTCCAAACTCTTGCCTCAAAG-3′ and reverse, 5′-CCAGCCGGGTTTTGGACG-3′. The products were cloned into the pSilencer 4.1 cmv puro vector (Applied Biosystems, Waltham, Massachusetts, USA) between the BamH I and Hind III sites as pre-miR-193b-3p.

The whole length of human TGF-β2 3′UTR was amplified with the following primers: forward, 5′-CTAGCTAGCAATTCTTGGAAAAGTGGCAAGACC-3′and reverse, 5′-CTAGCTAGCACTAGACAAAGCAAGACAACCAGA-3′. The products were cloned into the pGL3 promoter vector (Promega, Madison, Wisconsin, USA) downstream of the Luciferase reporter gene in the Nhe I site as pGL3-TGF-β2.

Expression plasmids of pGL3-TGF-β2-mutation without the *miR-193b-3p* binding site(s) were constructed by sequence overlapped extension-PCR with the following mutation primer sequences: forward, 5′-CTTTTGAGTAAAGCCCCTATA-3′ and reverse, 5′-CATACATTTGTGAGTGATCATTA-3′. A 1,406-bp fragment of the TGF-β2 CDS region was amplified with PCR from the cDNA of HTR-8/SVneo cell line using primers in Supplementary Table S4. The PCR product was cloned into a pcDNA (Applied Biosystems, Waltham, Massachusetts, USA) using Xba I and Xho I restriction sites as the TGF-β2 expression vector pcDNA-TGFβ2.

### Cell culture and transfection

To investigate the effect of *miR-193b-3p* overexpression, HTR-8/SVneo cells were cultured in RPMI-1640 (Gibco, California, USA) on a 24-well plate and transfected with 800 ng of pre-miR-193b-3p plasmid or control constructs (negative control, NC pre-miR-193b-3p) per well. To investigate the effect of *miR-193b-3p* Inhibition, HTR-8/SVneo cells were cultured in RPMI-1640 (Gibco, California, USA) on a 24-well plate and transfected with 100 pmol *miR-193b-3p* inhibitor (GenePharma, China) or control constructs (negative control, NC Inhibitor) per well. To investigate the roles of TGF-β2 during the suppression of migration and invasion of trophoblast cells by *miR-193b-3p*, we co-transfected HTR8/SVneo cells with 200 ng pre-miR-193b-3p and 200 ng pcDNA-TGFβ2 to perform a rescue experiment. We also co-transfected HTR8/SVneo cells with 50 pmol *miR-193b-3p* inhibitor (GenePharma, China) and 50 pmol *TGF-β2* siRNA (GenePharma, China) to perform a rescue experiment. The sequence information of *miR-193b-3p* inhibitor (GenePharma, China) and *TGF-β2* siRNA (GenePharma, China) were described in Supplementary Table S4.

### *In vitro* scratch assay

An *in vitro* scratch assay was used as described previously^54^. The protocol of cell culture and transient transfection is described previously. When HTR-8/SVneo cells were grown to 90% confluence, the scratches were made. In order to minimize cell proliferation, the cells were allowed to grow in RPMI-1640 (Gibco, California, USA) without foetal bovine serum. The width of the scratch was monitored by LEICA DMI4000B within 24 h after transfection.

### Transwell migration assay

The protocol of cell culture and transient transfection is described previously. Transwell compartments were prepared with 24-well format and 8 μm pore size insert. For the lower compartment, we added 0.8 ml of RPMI-1640 (Gibco, California, USA) with 10% FBS; and for the upper compartment, we gently added 5 × 10^4^ cells in 100 μl serum-free RPMI-1640 (Gibco, California, USA). We incubated the cells in the transwell plate at 37 °C and 5% CO2 for 6 h. After 6 h, we carefully took the insert out. Cells on the upper surface of membranes were completely removed. and the cells migrated to the lower surface of membranes were fixed with 100% formaldehyde and stained with 0.05% crystal violet. The number of migrated cells was counted under LEICA DMI4000B. Cell migration level was presented as the percentage of migrated cell number compared with the corresponding control.

### Transwell invasion assay

The protocol of cell culture and transient transfection was described previously. We prepared for the transwell 24-well fitted inserts (8 μm pore size, Millipore Corp, Massachusetts, USA). The transwell inserts were pre-coated with 7 μg matrigel (BD Biosciences, NJ, USA) in 50 μl serum-free culture medium. For the lower compartment, we added 0.8 ml of RPMI-1640 (Gibco, California, USA) with 10% FBS. For the upper compartment, we gently added 5 × 10^4^ cells in 100 μl serum-free RPMI-1640 (Gibco, California, USA). We incubated the cells in the transwell plate at 37 °C and 5% CO2 for 24 h. After 24 h, we carefully took the insert out. Cells on the upper surface of membranes were completely removed, and the cells migrated to the lower surface of membranes were fixed with 100% formaldehyde and stained with 0.05% crystal violet. The number of invaded cells was counted under LEICA DMI4000B. Cell invasion level was presented as the percentage of invaded cell number compared with the corresponding control.

### Luciferase assays

Luciferase assays were performed with the Dual-Luciferase Reporter Assay System (Promega, Madison, Wisconsin, USA). pRL-SV40 (Promega, Madison, Wisconsin, USA), a control plasmid, was used to check for transfection efficiency. To investigate the effect of *miR-193b-3p* overexpression, 293 and HTR-8/SVneo cells were cotransfected with luciferase reporter plasmids (300 ng), pRL-SV40 (15 ng) and pre-miR-193b-3p expression vector (500 ng) or control constructs NC pre-miR-193b-3p (500 ng) per well. To investigate the effect of *miR-193b-3p* inhibition, 293 and HTR-8/SVneo cells were cotransfected with luciferase reporter plasmids (300 ng), pRL-SV40 (15 ng) and *miR-193b-3p* Inhibitor (100 pmol) or control NC Inhibitor (100 pmol).

### Western blotting

Placental tissues were homogenized in lysis buffer with added proteinase inhibitors (Roche, Penzberg, Germany). A BCA kit (Thermo Fisher Scientific, Waltham, Massachusetts, USA) was used to assess protein concentrations. 20 ug of proteins were resolved by 10% SDS-PAGE and transferred to a nitrocellulose membrane (Applied Biosystems, Waltham, Massachusetts, USA). The membranes were incubated with the primary antibodies against TGF-β2 (Abcam, Cambridge, UK, 1:500) and GAPDH (Abmart, Shanghai, China, 1:1000) overnight at 4 °C. Following this, the membranes were incubated with horseradish peroxidase (HRP) conjugated secondary antibodies (Abmart, Shanghai, China, 1:3000). The signal intensity was quantitatively assessed with Fujifilm LAS-3000 Imager and was normalized to GAPDH.

### Statistical analysis

Analysis of microRNA-seq data was performed using R version 2.13.1. A 2-tailed t test was performed; and Mann-Whitney test was performed with SPSS 17 (SPSS Inc., Chicago, Illinois, USA) when the data pattern did not follow Gaussian distribution. The results are shown as the mean ± S.E.M. or mean ± S.D. A threshold of P value <0.05 was considered to be significant. Correlation was calculated using Pearson correlation coefficient.

## Additional Information

**How to cite this article**: Zhou, X. *et al.* The aberrantly expressed miR-193b-3p contributes to preeclampsia through regulating transforming growth factor-β signalling. *Sci. Rep.* **6**, 19910; doi: 10.1038/srep19910 (2016).

## Supplementary Material

Supplementary Information

## Figures and Tables

**Table 1:** Differentially regulated microRNAs in preeclamptic placenta compared to normal placenta.

MicroRNA Name	Fold Change	p-value
*miR-10b-5p*	3.07	0.002
*miR-210*	2.70	<0.001
*miR-148a-3p*	2.63	0.002
*miR-148a-5p*	2.46	0.001
*miR-193b-5p*	2.30	0.001
*miR-9-5p*	2.28	0.006
*miR-520a-3p*	2.19	0.004
*miR-151a-3p*	2.06	0.007
*miR-193b-3p*	2.01	0.003
*miR-192-5p*	1.98	0.007
*miR-520a-5p*	1.96	0.010
*miR-31-5p*	1.95	0.006
*miR-365a-3p*	1.88	0.010
*miR-365b-3p*	1.88	0.010
*miR-516b-5p*	1.87	0.010
*miR-5695*	1.85	0.010
*miR-27a-5p*	1.77	0.009
*miR-136-3p*	0.58	0.010
*miR-874*	0.56	0.010
*miR-382-3p*	0.53	0.006
*miR-539-3p*	0.51	0.002
*miR-584-5p*	0.50	0.003
*miR-889*	0.49	0.001
*miR-34b-5p*	0.48	0.002
*miR-135b-5p*	0.46	<0.001

**Table 2:** Clinical characteristics of subjects with normal and preeclamptic pregnancies.

	Initial discovery set(HTS)			Validation set		
	Control (n = 9)	Preeclampsia (n = 9)	p value	Control (n = 20)	Preeclampsia (n = 22)	P value
Maternal age (y)	28.3 ± 1.4	32.1 ± 6.9	0.16	30.5 ± 4.4	30.4 ± 4.7	0.329
Gestational age (days)	280.3 ± 3.9	244.3 ± 21.3	<0.01	277.1 ± 7.2	232.5 ± 25.2	<0.01
Birthweight (g)	3694.4 ± 584.2	2243 ± 941.1	<0.01	3502.9 ± 395.9	1991.8 ± 966.2	<0.01
BMI (kg/m^2^)	29.5 ± 3.2	30.8 ± 3.2	0.23	29.5 ± 3.1	31.0 ± 5.9	0.45
Proteinuria (g/24 h)	Not detected	3.3 ± 3.5	<0.01	Not detected	4.7 ± 3.2	<0.01
SBP (mm/Hg)	120.9 ± 19.7	161.5 ± 18.2	<0.01	123.6 ± 11.0	164.1 ± 23.5	<0.01
DBP (mm/Hg)	79.0 ± 10.0	109.0 ± 12.4	<0.01	79.5 ± 9.3	112.4 ± 16.6	<0.01
