# Supplementary material for: The aberrantly expressed miR-193b-3p contributes to preeclampsia through regulating transforming growth factor-β signaling
Source: Sci Rep. 2016 Jan 29;6:19910. doi: 10.1038/srep19910 (PMC4731805; doi:10.1038/srep19910)
Supplement: Supplementary Information [file srep19910-s1.pdf]

Supplementary information

The aberrantly expressed miR-193b-3p contributes to preeclampsia through regulating transforming growth factor-β signalling

Xinyao Zhou[1, *], Qiaoli Li[1, *], Jiawei Xu[1], Xiaojing Zhang[3], Huijuan Zhang[4], Yuqian Xiang[1], Chuantao Fang[1], Teng Wang[1], Shihui Xia[1], Qiang Zhang[1], Qinghe Xing[1], Lin He[5], Lei Wang[2, +], Mingqing Xu[5, +], Xinzhi Zhao [1, +]

[1] Children's Hospital and Institutes of Biomedical Sciences, Fudan University, 138 Yixueyuan Road, Shanghai 200032 , China

[2] State Key Laboratory of Genetic Engineering and MOE Key Laboratory of Contemporary Anthropology, School of Life Sciences, Fudan University, Shanghai, China

[3] Department of Obstetrics, Provincial Hospital Affiliated to Shandong University, Jinan, China

[4] Departments of Pathology and Biobank, International Peace Maternity & Child Health Hospital of China affiliated to Shanghai Jiao Tong University, Shanghai, China

[5] Bio-X Institutes, Key Laboratory for the Genetics of Developmental and Neuropsychiatric Disorders (Ministry of Education), Shanghai Jiao Tong University, Shanghai, China

* These authors contributed equally to this work.

+ Correspondence and requests for materials should be addressed to X.Z. (email: xzzhao@fudan.edu.cn), M.X. (email: mingqingxu@gmail.com) or L.W. (email: wangleiwanglei@fudan.edu.cn)

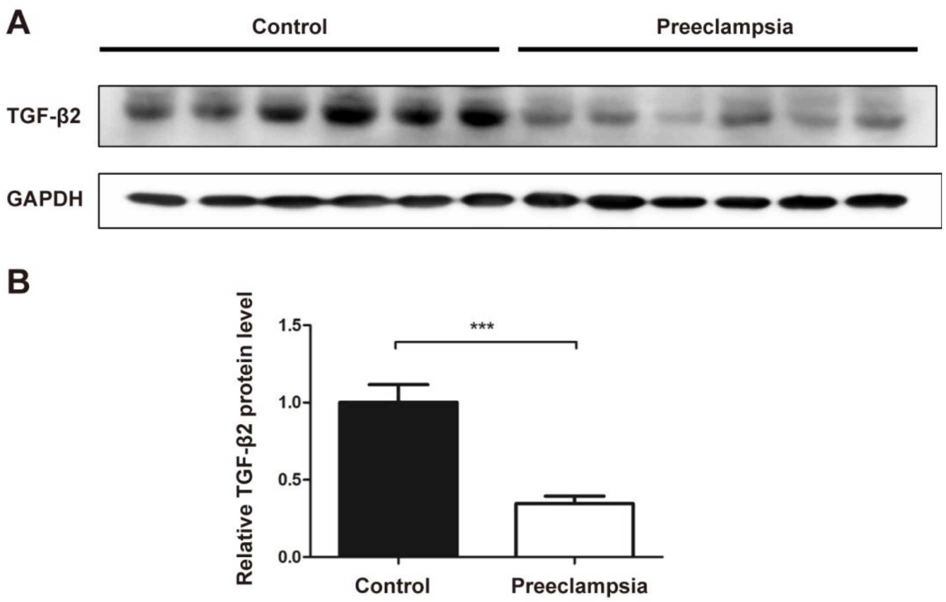

Supplementary Figure S1 TGF-β2 protein level in control and preeclamptic placentas Western blot for TGF-β2 detection of protein extracts from control (n=6) and preeclamptic (n=6) placental tissues (**A**). GAPDH was used as the loading control, and the density of the TGF-β2 band was assessed relative to GAPDH (**B**).

Supplementary Table S1 The percentage of expression of the top 30 microRNAs in normal and preeclamptic placenta

**MicroRNA Name**	**Normal Placenta** (percentage of all microRNAs)	**Preeclamptic Placenta** (percentage of all microRNAs)
miR-143-3p	26.4351	20.6924
miR-26a-5p	11.8147	10.6042
miR-181a-5p	4.9986	6.4929
miR-22-3p	3.7572	3.5086
miR-30a-5p	3.3244	3.5993
miR-27b-3p	3.1240	2.7602
miR-486-5p	3.0861	4.6052
miR-30d-5p	2.0264	1.6158
miR-99b-5p	1.9068	1.3661
miR-100-5p	1.8721	1.3510
miR-141-3p	1.8616	1.6668
miR-191-5p	1.6053	1.7621
miR-10b-5p	1.5158	4.4187
miR-127-3p	1.4716	0.9332
miR-10a-5p	1.3912	1.4070
miR-21-5p	1.3677	1.7085
miR-126-5p	1.2470	1.1828
miR-451a	1.0932	0.8199
miR-125a-5p	1.0776	0.8899
let-7a-5p	0.8564	0.8830
miR-516a-5p	0.8284	1.0428
miR-518b	0.7875	1.1452
miR-27a-3p	0.7782	0.7864
miR-26b-5p	0.7086	0.6494
miR-516b-5p	0.6894	1.2242
miR-517a-3p	0.6790	0.6728
miR-517b-3p	0.6790	0.6728
miR-30b-5p	0.6198	#N/A
miR-512-3p	0.5887	0.8173
miR-126-3p	0.5333	#N/A
miR-148a-3p	#N/A	1.2925
miR-92a-3p	#N/A	0.6798
Other	17.2754	18.7492

Supplementary Table S2 Consistent results of differentially expressed microRNAs in the present study and previous studies of PE placenta

MicroRNA Name	Present in number of publications	Expression in preeclampsia	References
miR-210	8	Up	Zhang 2014[1], Xu 2014[2], Betoni 2013[3], Ishibashi 2012[4], Enquobahrie 2011[5], Zhu 2009[6], Pineles 2007[7], **Current Study**
miR-193b-3p	4	Up	Xu 2014, Betoni 2013, Ishibashi 2012, **Current Study**
miR-181a-5p	4	Up	Zhang 2014, Xu 2014, Zhu 2009, Hu 2009[8]
miR-296-5p	3	Up	Xu 2014, Choi 2013[9], Zhu 2009
miR-519e-5p	3	Up	Xu 2014, Ishibashi 2012, Zhu 2009
miR-16-5p	2	Up	Zhang 2014, Hu 2009
miR-20b-5p	2	Up	Wang 2012[10], Hu 2009
miR-30a-3p	2	Up	Xu 2014, Zhu 2009
miR-31-5p	2	Up	Xu 2014, **Current Study**
miR-141-3p	2	Up	Guo 2011[11], Hu 2009
miR-182-3p	2	Up	Noack 2011[12], Pineles 2007
miR-182-5p	2	Up	Zhang 2014, Pineles 2007
miR-193b-5p	2	Up	Ishibashi 2012, **Current Study**
miR-362-5p	2	Up	Xu 2014, Zhu 2009
miR-451a	2	Up	Ishibashi 2012, Hu 2009
miR-517c-3p	2	Up	Ishibashi 2012, Guo 2011
miR-520-3p	2	Up	Ishibashi 2012, **Current Study**
miR-524-3p	2	Up	Xu 2014, Wang 2012
miR-638	2	Up	Xu 2014, Zhu 2009
miR-10b-5p	2 vs. 1[#]	Up	Ishibashi 2012, Zhu 2009, **Current study**
miR-20a-5p	2 vs. 1	Up	Zhang 2014, Betoni 2013, Ishibashi 2012
miR-26b-5p	2 vs. 1	Up	Choi 2013, Betoni 2013, Hu 2009
miR-126-5p	2 vs. 1	Up	Ishibashi 2012, Guo 2011, Zhu 2009
miR-151-3p	2 vs. 1	Up	Xu 2014, Wang 2012, **Current Study**
miR-363-3p	4	Down	Zhang 2014, Xu 2014, Betoni 2013, Zhu 2009

miR-218-5p	3	Down	Xu 2014, Betoni 2013, Zhu 2009
miR-542-3p	3	Down	Xu 2014, Betoni 2013, Zhu 2009
miR-30d-5p	2	Down	Mayar-Lynn 2011[13], Guo 2011
miR-34c-5p	2	Down	Wang 2012, Enquobahrie 2011
miR-101-3p	2	Down	Betoni 2013, Zhu 2009
miR-145-3p	2	Down	Betoni 2013, Mayar-Lynn 2011
miR-146a-5p	2	Down	Wang 2012, Mayar-Lynn 2011
miR-214-3p	2	Down	Xu 2014, Hu 2009
miR-224-5p	2	Down	Betoni 2013, Ishibashi 2012
miR-411-5p	2	Down	Xu 2014, Zhu 2009
miR-223-3p	4 vs. 2[&]	Down	Xu 2014, Choi 2013, Betoni 2013, Guo 2011, Mayar-Lynn 2011, Zhu 2009
miR-584-5p	3 vs. 2	Down	Zhang 2014, Xu 2014, Enquobahrie 2011，Zhu 2009，**Current Study**
miR-1	2 vs. 1	Down	Zhang 2014, Enquobahrie 2011, Zhu 2009
miR-18a-5p	2 vs. 1	Down	Xu 2014, Ishibashi 2012, Zhu 2009
miR-19a-3p	2 vs. 1	Down	Xu 2014, Ishibashi 2012, Zhu 2009
miR-192-5p	2 vs. 1	Down	Betoni 2013, Wang 2012, **Current Study**
miR-195-5p	2 vs. 1	Down	Xu 2014, Hu 2009, Zhu 2009
miR-590-5p	2 vs. 1	Down	Xu 2014, Ishibashi 2012, Zhu 2009
miR-518b	2 vs. 2[§]	-	Xu 2014, Guo 2011, Mayar-Lynn 2011, Zhu 2009

The publication marked in bold indicates that the corresponding microRNA was found to be differentially expressed in the present high-throughput study.

The microRNAs marked in bold were found to be differentially expressed in the present high-throughput study, and the current result is consistent with at least one previous study.

[#] This microRNA is present in 3 studies, and 2 studies reported consistent results.

[&] This microRNA is present in 6 studies, and 4 studies reported consistent results.

[§]This microRNA is present in 4 studies; 2 studies reported consistent results, and the remaining two studies had the opposite result.

Supplementary Table S3 Detailed clinical characteristics of patients with normal and preeclamptic pregnancies in HTS study

Sample ID	Maternal age (y)	Gestational age (days)	Birthweight (g)	BMI (kg/m2)	Proteinuria (g/24 h)	SBP (mm/Hg)	DBP (mm/Hg)
C1	29	283	3700	25.91	0	124	75
C2	28	277	4400	27.06	0	126	88
C3	31	278	3140	25.64	0	105	61
C4	29	277	3100	31.64	0	130	89
C5	27	285	2820	35.25	0	126	88
C6	28	275	4410	29.14	0	128	74
C7	28	286	3580	28.63	0	125	89
C8	26	279	3930	29.41	0	120	76
C9	29	283	4170	32.69	0	104	71
P1	26	271	3970	30.84	6.7	164	114
P2	25	230	1460	36.05	11.27	161	126
P3	22	264	2740	34.53	1.2	158	87
P4	35	218	1080	31.57	2.0	147	119
P5	39	244	2200	30.12	5.1	157	109
P6	35	218	1100	26.17	0.3	156	106
P7	42	224	1600	30.78	2.0	165	106
P8	36	262	2980	28.98	1.0	210	125
P9	25	242	2350	32.41	2.37	150	100

C1-C9, normal placentas; and P1-P10, preeclamptic placentas.
BMI, indicates body mass index; DBP, diastolic blood pressure; and SBP, systolic blood pressure.

Supplementary Table S4 microRNA and primer sequence

miR-148a-3p	microRNA mature sequence	UCAGUGCACUACAGAACUUUGU
	RT primer	GTTGGCTCTGGTGCAGGGTCCGAGGTATTCGCACCAGAGCCAACACAAAG
	Forward primer	CCGCCGATCAGTGCACTACAGAA
miR-210	microRNA mature sequence	CUGUGCGUGUGACAGCGGCUGA
	RT primer	GTTGGCTCTGGTGCAGGGTCCGAGGTATTCGCACCAGAGCCAACTCAGCC
	Forward primer	CGCGGCGCCTGTGCGTGTGACAGC
miR-193b-3p	microRNA mature sequence	AACUGGCCCUCAAAGUCCCGCU
	RT primer	GTTGGCTCTGGTGCAGGGTCCGAGGTATTCGCACCAGAGCCAACAGCGGG
	Forward primer	CCGCCGAAACTGGCCCTCAAAGT
miR-31-5p	microRNA mature sequence	AGGCAAGAUGCUGGCAUAGCU
	RT primer	GTTGGCTCTGGTGCAGGGTCCGAGGTATTCGCACCAGAGCCAACAGCTAT
	Forward primer	TCTATGAGGCAAGATGCTGGC
miR-365a-3p	microRNA mature sequence	UAAUGCCCCUAAAAAUCCUUAU
	RT primer	GTTGGCTCTGGTGCAGGGTCCGAGGTATTCGCACCAGAGCCAACATAAGGA
	Forward primer	CGCACGTAATGCCCCTAAAAAT
miR-516b-5p	microRNA mature sequence	AUCUGGAGGUAAGAAGCACUUU
	RT primer	GTTGGCTCTGGTGCAGGGTCCGAGGTATTCGCACCAGAGCCAACAAAGTG
	Forward primer	TCTATGATCTGGAGGTAAGAAG
miR-520a-5p	microRNA mature sequence	CUCCAGAGGGAAGUACUUUCU
	RT primer	GTTGGCTCTGGTGCAGGGTCCGAGGTATTCGCACCAGAGCCAACAGAAAGT
	Forward primer	GGTCAACTCCAGAGGGAAGTA
miR-27a-5p	microRNA mature sequence	AGGGCUUAGCUGCUUGUGAGCA
	RT primer	GTTGGCTCTGGTGCAGGGTCCGAGGTATTCGCACCAGAGCCAACTGCTCA
	Forward primer	TCTATGAGGGCTTAGCTGCTTG
miR-135b-5p	microRNA mature sequence	UAUGGCUUUUCAUUCCUAUGUGA
	RT primer	GTTGGCTCTGGTGCAGGGTCCGAGGTATTCGCACCAGAGCCAACTCACAT

	Forward primer	CGCGGCGCTATGGCTTTTCATTCC
miR-136-3p	microRNA mature sequence	CAUCAUCGUCUCAAAUGAGUCU
	RT primer	GTTGGCTCTGGTGCAGGGTCCGAGGTATTCGCACCAGAGCCAACAGACTC
	Forward primer	TCTATGCATCATCGTCTCAAAT
*TGF-β*2	qRT-PCR Forward primer	CAGCACACTCGATATGGACCA
	qRT-PCR Reverse primer	GTTGTAGATGGAAATCACCTCCG
*TGF-β*2 siRNA	Sense	GACCCUACUUCAGAAUUGUTT
	Antisense	ACAAUUCUGAAGUAGGGUCTT
miR-193b-3p inhibitor	Sense	AACUGGCCCUCAAAGUCCCGCU
	Antisense	AGCGGGACUUUGAGGGCCAGUU
pcDNA-TGFβ2	CDS Forward primer	GCTCTAGAATGCACTACTGTGTGCTGA
	CDS Reverse primer	CCGCTCGAGACAAGCATCATCGTTGTCG

Reference

1 Zhang, C. et al. Placental miR-106a approximately 363 cluster is dysregulated in preeclamptic placenta. *Placenta.* **36**, 250-252, (2015).

2 Xu, P. *et al.* Variations of microRNAs in human placentas and plasma from preeclamptic pregnancy. *Hypertension.* **63**, 1276-1284, (2014).

3 Betoni, J. S. *et al.* MicroRNA analysis in placentas from patients with preeclampsia: comparison of new and published results. *Hypertens Pregnancy.* **32**, 321-339, (2013).

4 Ishibashi, O. *et al.* Hydroxysteroid (17-beta) dehydrogenase 1 is dysregulated by miR-210 and miR-518c that are aberrantly expressed in preeclamptic placentas: a novel marker for predicting preeclampsia. *Hypertension.* **59**, 265-273, (2012).

5 Enquobahrie, D. A. *et al.* Placental microRNA expression in pregnancies complicated by preeclampsia. *Am J Obstet Gynecol.* **204**, 178 e112-121, (2011).

6 Zhu, X. M., Han, T., Sargent, I. L., Yin, G. W. & Yao, Y. Q. Differential expression profile of microRNAs in human placentas from preeclamptic pregnancies vs normal pregnancies. *Am J Obstet Gynecol.* **200**, 661 e661-667, (2009).

7 Pineles, B. L. *et al.* Distinct subsets of microRNAs are expressed differentially in the human placentas of patients with preeclampsia. *Am J Obstet Gynecol.* **196**, 261 e261-266, (2007).

8 Hu, Y. *et al.* Differential expression of microRNAs in the placentae of Chinese patients with severe pre-eclampsia. *Clin Chem Lab Med.* **47**, 923-929, (2009).

9 Choi, S. Y. *et al.* MicroRNA expression profiles in placenta with severe preeclampsia using a PNA-based microarray. *Placenta.* **34**, 799-804, (2013).

10 Wang, W. *et al.* Preeclampsia up-regulates Angiogenesis-associated microRNA (i.e., miR-17, -20a, and -20b) that target Ephrin-B2 and EPHB4 in human placenta. *J Clin Endocrinol Metab.* **97**, E1051-E1059, (2012).

11 Martelli, F. *et al.* A Comprehensive Survey of miRNA repertoire and 3′ addition events in the placentas of patients with pre-Eclampsia from high-throughput sequencing. *PLoS One.***6**, e21072, (2011).

12 Noack, F. *et al.* miRNA expression profiling in formalin-fixed and paraffin-embedded placental tissue samples from pregnancies with severe preeclampsia. *J Perinat Med.* **39**, 267-271, (2011).

13 Mayor-Lynn, K., Toloubeydokhti, T., Cruz, A. C. & Chegini, N. Expression profile of microRNAs and mRNAs in human placentas from pregnancies complicated by preeclampsia and preterm labor. *Reprod Sci.* **18**, 46-56, (2011).
